# Association of dietary patterns with general and central obesity among Chinese adults: a longitudinal population-based study

**DOI:** 10.1186/s12889-023-16556-0

**Published:** 2023-08-21

**Authors:** Zhongyi Zhao, Shihan Zhen, Yumeng Yan, Ning Liu, Ding Ding, Juan Kong

**Affiliations:** 1 Department of Clinical Nutrition, Shengjing Hospital of China Medical University, Shenyang, 110004 China; 2 Department of Health Management, Shengjing Hospital of China Medical University, Shenyang, 110004 China; 3 School of Public Health, China Medical University, Shenyang, 110122 China

**Keywords:** Dietary pattern, General obesity, Central obesity, Longitudinal, CHNS

## Abstract

**Background:**

Limited evidence exists for the association between dietary patterns and later obesity phenotypes among Chinese adults. This longitudinal study aimed to evaluate associations of dietary patterns with general and central obesity in Chinese adults.

**Methods:**

Based on the China Health and Nutrition Survey (CHNS) waves 2004 and 2015, the study was conducted on 4207 adult men and women (age range: 18–65 years). Dietary intakes were assessed by three consecutive 24-h dietary recalls, and dietary patterns were identified using exploratory factor analysis. Longitudinal associations of dietary patterns with general and central obesity were evaluated using logistic regression analyses.

**Results:**

The prevalence rates of general and central obesity were 14.2% and 42.1%, respectively. Factor analysis extracted three major dietary patterns: “traditional southern,“ “modern,“ and “traditional northern.“ After adjustment for potential confounders, adults in the highest quartile of the traditional southern dietary group were less likely to develop over 10 years general (odds ratio [OR] = 0.50, 95% confidence interval [95%CI]: 0.39, 0.65) and central (OR = 0.52, 95%CI: 0.43, 0.63) obesity compared to those in the lowest quartile group. The modern dietary pattern was not significantly associated with general and central obesity. Adherence to the traditional northern dietary pattern increased the chance of both general and central obesity (OR = 1.61, 95%CI: 1.23, 2.10; OR = 1.64, 95%CI: 1.36, 1.98) after 10 years.

**Conclusions:**

Our study provides longitudinal evidence for associations between dietary patterns and later obesity phenotypes among Chinese adults. Our findings may guide the development of evidence-based preventive nutrition interventions to control the obesity epidemic.

## Background

Obesity has become a major global public health problem, and its prevalence has nearly tripled in recent decades [1, 2]. According to the global Nutrition Report 2021, the number of obese adults in the world is as high as 772 million, and more than 40% of adults are overweight or obese [3]. In China, the prevalence rates of general obesity and central obesity among adults of all age and gender groups have increased significantly over the past 20 years [4]. Obesity has a negative impact on almost all physiological functions of the body and is a major risk factor associated with numerous noncommunicable diseases [5]. Moreover, central obesity, defined by waist circumference (WC), is an independent predictor of all-cause mortality [6].

It is well known that obesity is a complicated multifactorial chronic disorder with multiple causal factors, including genetic and environmental. Of these, dietary factors are believed to play a key role and have a well-established influence on the risk of obesity and related metabolic diseases [7]. Although dietary factors have been attributed to the development of obesity, the relationship is complex and not fully understood [8]. Compared with traditional dietary analyses, dietary patterns represent a combination of food and nutrients consumption and have gradually become the preferred method for determining individual dietary intake and clarifying dietary behavior [9]. Posteriori dietary pattern analysis is more appropriate and predictive than the analysis of individual food or nutrient components in studying the relationship between diet and chronic diseases [10]. Several previous studies have reported links between distinct dietary patterns with general and central obesity [11–14], and the dietary patterns vary widely across countries, cultures, and ethnic groups. In general, dietary patterns with higher intakes of vegetables, fruits, high-fiber grains, and fish are thought to be inversely associated with obesity, whereas dietary patterns with higher intakes of red meat, processed meat, sweets, and fast food are considered to be positively correlated with obesity [15].

China, a developing country, is undergoing a huge transformation in dietary structure and nutrition due to rapid social and economic developments [16, 17]. The traditional Chinese diet, which is characterized by high carbohydrate intake including rice, wheat, and cooked vegetables, is shifting to a diet with increased consumption of animal-based food and other energy-rich food components [18]. Westernization of dietary habits may affect the prevalence of chronic diseases such as obesity in China [19]. Therefore, it is necessary to study dietary patterns and their relationships with obesity regarding the specifics of the Chinese culture. To the best of our knowledge, most previous studies on dietary patterns and obesity have followed a cross-sectional design [20–22]. In particular, there is a lack of evidence for the long-term associations of different dietary patterns on obesity phenotypes among Chinese adults. Therefore, in the present longitudinal study, we aimed to characterize dietary patterns at baseline and to examine the associations between dietary patterns with later general and central obesity in a large sample of Chinese adults.

## Methods

### Study design and population

We used data collected in the China Health and Nutrition Survey (CHNS), an ongoing large-scale, longitudinal, household-based survey initiated in 1989. The CHNS used a multistage random-cluster process to draw the sample and covered 15 provinces including Heilongjiang, Liaoning, Beijing, Henan, Jiangsu, Zhejiang, Shanghai, Shandong, Hubei, Hunan, Shaanxi, Yunnan, Chongqing, Guizhou, and Guangxi that varied in demography, geography, economic development, and public resources [23]. In each province, we selected 2 cities and 4 counties by income level, and randomly selected 4 communities in each city or county. Follow-up investigations were conducted every 2–4 years for the same population, with demographic, socio-economic, lifestyle, nutritional and health data collected in each wave. Additional details about the CHNS data are provided elsewhere [24]. Survey protocols, instruments, and the process for obtaining informed consent for the CHNS were approved by the Institutional Review Committees of the University of North Carolina at Chapel Hill and the National Institute of Nutrition and Food Safety, China Centre for Disease Control and Prevention. Written informed consent was obtained from each participant.

We used the longitudinal CHNS data from 2004 to 2015 wave. Data derived from the 2004 wave was analyzed as baseline and included 8377 Chinese adult participants aged 18–65 years with complete information on dietary assessment. We further excluded participates with missing anthropometric data at 2015 follow-up (*n* = 4130), with extreme daily energy intakes (< 800 kcal or > 6000 kcal for men and < 600 kcal or > 4000 kcal for women, *n* = 15) in 2004, and women who were pregnant in either wave of survey (*n* = 25). Finally, 4207 participants were selected in the present study.

### Dietary assessment and food grouping

At baseline, the dietary assessment was based on a combination of three consecutive 24-h dietary recalls at the individual level, as well as a household food inventory taken during the same 3-day period. The three consecutive days during which detailed food consumption data have been collected were randomly allocated to start from Monday to Sunday. Previous studies have provided details of dietary measurements in the CHNS study [25]. The food groups included were based on a food grouping system developed specifically for the CHNS [26]. We consolidated the categories that consumed less than 5% of the original food groups. Finally, food groups were collapsed into 21 food groups based on similar nutrient profiles, culinary use, or the grouping scheme used in other studies (see Table 1). Total energy intakes were computed using the Chinese Food Composition Tables [27, 28].

Exploratory factor analysis using principal component analysis was used to identify dietary patterns at baseline (2004). We used the estimated intakes (g/d) of 21 food groups as the input values in factor analyses. To improve interpretability, factors were rotated by orthogonal (varimax) rotation to minimize correlations between factors. When considering the retained factors, we evaluated the eigenvalue (> 1), scree plot, factor interpretability, and variance explained (> 5%) to determine which set of factors best described the different dietary patterns. Items remained in a factor if they had an absolute correlation of > 0.20 with that pattern [29]. The cumulative variance of the retained factors explains the variation in food intake. Labeling of the factors was primarily descriptive based on the interpretation of our data, and the factor scores for each pattern were divided into four quartiles (the lowest quartile to the highest quartile, Q1 to Q4) for further analysis.

**Table 1:** Food groups used in the factor analysis

Food group	Food items included in the group
Rice	White and brown rice
Wheat flour	Wheat flour
Wheat buns, breads	Bun, butter bread, salty bread
Deep-fried wheat	Deep-fried dough stick, deep-fried cake with red bean paste and sugar, deep-fried sweet sesame seed ball
Corn and coarse grain	Corn, corn grits, corn flour, barley, oats, foxtail millet, sorghum
Starchy roots and tubers	Potato, yam, taro, lotus root, water chestnut, cassava, sweet potato
Fresh legumes	Soybean sprouts, peas in pods, mung bean sprouts
Dried legumes	Soybean flour, dried beans, bean flour, roasted broad beans
Legume products	Tofu, tofu products, red/mung bean paste
Nuts and seeds	Sesame, sunflower, watermelon, and lotus seeds, peanuts, walnuts, almonds, hazelnuts, pinenuts, pistachios, cashew nuts
Fresh vegetables	Spinach, bok choy, cabbage, cauliflower, tomatoes, cucumber, zucchini, mushrooms
Pickled, salted, or canned vegetables	Canned tomato sauce, preserved vegetables, vegetables in soy sauce
Fruits	Fresh and canned fruits (without added sugar), dates, dried longan, raisins, dried and canned fruit (with added sugar)
Pork	Pork tenderloin, pork tendons, pork belly, leg, rib chop
Other livestock meat	Beef, lamb, donkey, rabbit
Offal	Liver, kidney, large intestine, blood
Poultry and game	Chicken, duck, goose
Eggs and egg products	Whole eggs, yolk, egg white, preserved eggs
Fish and seafood	Fresh- and salt-water fish, dried fish, shellfish
Animal-based milk and dairy products	Cow milk, goat milk, skim milk, flavored milk, cheese, yogurt, ice cream
Alcoholic beverages	Liquors, wine, vodka, cocktails, whiskey, beer

### Outcome variables

Anthropometric measures of participants wearing light clothing and no shoes were assessed by trained technicians who followed standard protocols recommended by the World Health Organization (WHO) [30]. Weight was measured to the nearest 0.1 kg, and height was measured to the nearest 0.1 cm. Waist circumference was measured at a level halfway between the lower rib cage and iliac crest during exhalation. Body mass index (BMI) was calculated as weight in kilograms divided by the square of height in meters. General obesity was defined as BMI ≥ 28 kg/m2, based on the criteria recommended by Working Group on Obesity in China (WGOC) [31]. Central obesity was defined as WC ≥ 90 cm for men and WC ≥ 85 for women [32].

### Covariates

Participants completed a questionnaire designed to assess their demographic and lifestyle data such as age (year), gender (man or woman), ethnicity (Han Chinese or minority), education level (illiteracy, primary school, middle school, and college or above), marital status (married or other), work status (employed or unemployed), residency (urban or rural area), total energy intake per day (kcal/d), and sleep duration (≤ 7 h, 7–9 h, and ≥ 9 h). We calculated the Metabolic Equivalent of Task (MET) based on the “Compendium of Physical Activities,” including four types of physical activities: domestic, occupational, transportation, and leisure physical activity during the past 12 months. The level of physical activity was the product of time spent in each activity multiplied by specific MET values [33, 34]. Current smoking was used to assess smoking events, and those who had consumed beer, liquor, or other alcoholic beverages in the past year were considered alcohol drinkers.

### Statistical analysis

The convergence and dispersion trends of quantitative variables were expressed as the mean ± standard deviation, whereas qualitative variables were expressed as frequency and percentage. Missing values of covariates were imputed using multiple imputation techniques [35]. The prevalence rates of general obesity and central obesity were calculated. We determined the sociodemographic variables of participants by gender. Continuous variables were compared using the independent sample t-test, and categorical variables were compared using Pearson’s χ2 test.

We assessed several logistic regression models to determine the association of dietary patterns at baseline with subsequent occurrences of general and central obesity. The likelihood ratio test was performed to test the logistic model assumption and fitness, and no violations were found. We first fitted a logistic model without controlling for any confounders (model 1). Model 2 controlled for age (in years) and gender (reference group: women). In model 3, we further adjusted for a series of covariates related to personal characteristics: ethnicity (reference group: Han nationality), education level (reference group: illiteracy), marital status (reference group: married), work status (reference group: unemployed), and residency (reference group: rural area). In model 4, we additionally introduced some confounders related to lifestyles: physical activity (in MET-h/week), sleep duration (reference group: ≤7 h), current smoking (reference group: no smoking), alcohol consumption (reference group: no alcohol consumption), and total energy intake per day (in kcal).

All statistical analyses were performed using Stata software (version 13.0) [36]. Statistical significance was set at *P* < 0.05 (two-sided).

## Results

Sample characteristics of the study population are presented in Table 2. Of 4207 participants, 1955 were men (46.5%), and 2252 were women (53.5%). The mean age of participants was 45.0 ± 10.8 years, with no difference between men and women (*P* = 0.123).

Men generally had a higher level of education (65.9% vs. 45.6% for middle school and above) than women. Compared to women, men had higher total energy intake (*P* < 0.001), higher levels of physical activity (*P* = 0.019), and higher rates of smoking and drinking (*P* < 0.001). In addition, the present study showed that 14.2% and 42.1% of participants had general and central obesity, respectively.

**Table 2:** Baseline characteristics of study participants in CHNS 2004 and anthropometric measures in 2015 by gender (*N* = 4207)

	Men	Women	*P* value
	1955 (46.5%)	2252 (53.5%)	
Age (years, mean ± SD)	44.8 ± 11.2	45.3 ± 10.5	0.123
Ethnicity (N, %)			0.876
Han Chinese	1687 (86.3)	1947 (86.5)	
Minority	268 (13.7)	305 (13.5)	
Education level (N, %)^a^			< 0.001
Illiteracy	157 (8.1)	587 (26.3)	
Primary school	503 (26.0)	630 (28.2)	
Middle school	1216 (62.9)	982 (43.9)	
College or above	58 (3.0)	37 (1.7)	
Marital status (N, %)^b^			< 0.001
Married	1718 (89.0)	2073 (92.9)	
Other	213 (11.0)	158 (7.1)	
Work status (N, %)^c^			< 0.001
Employed	1525 (78.9)	1380 (61.8)	
Unemployed	408 (21.1)	853 (38.2)	
Residency (N, %)			0.984
Urban area	529 (27.1)	610 (27.1)	
Rural area	1426 (72.9)	1642 (72.9)	
Total energy (kcal/d, mean ± SD)	2449.2 ± 682.8	2086.6 ± 567.3	< 0.001
Physical activity (MET-h/week, mean ± SD)	148.2 ± 129.0	139.3 ± 114.2	0.019
Sleep duration (N, %)^d^			< 0.001
≤7 h	498 (26.3)	466 (21.2)	
7–9 h	903 (47.6)	1040 (47.3)	
≥9 h	496 (26.2)	695 (31.6)	
BMI (kg/m^2^)	24.2 ± 4.5	24.4 ± 4.1	0.091
WC (cm)	85.9 ± 12.9	83.1 ± 12.7	< 0.001
General obesity (N, %)			0.031
Yes	254 (13.0)	345 (15.3)	
No	1701 (87.0)	1907 (84.7)	
Central obesity (N, %)			< 0.001
Yes	755 (38.6)	1016 (45.1)	
No	1200 (61.4)	1236 (54.9)	
Current smoking (N, %)			< 0.001
Yes	1178 (60.3)	76 (3.4)	
No	777 (39.7)	2176 (96.6)	
Alcohol consumption (N, %)			< 0.001
Yes	1249 (63.9)	194 (8.6)	
No	706 (36.1)	2058 (91.4)	

*SD *Standard deviation, *MET *Metabolic Equivalent of Energy, *BMI*, Body mass index, *WC *Waist circumference

Sample sizes are ^a^4170, ^b^4162, ^c^4166, and ^d^4098

Three major dietary patterns were identified from 21 predefined food groups at baseline (in 2004), and associated factor loading scores with absolute value > 0.20 are shown in Table 3. The first dietary pattern was defined as “traditional southern,“ which was represented by high consumption of rice, fresh legumes, fresh vegetables, pork, fish, and seafood, and a low intake of wheat, corn, and coarse grain. The second dietary pattern, “modern,“ was loaded heavily on fruits, poultry or game, eggs or egg products, and animal-based milk and dairy products. The third dietary pattern named “traditional northern” was rich in wheat buns, breads, starchy roots and tubers, fresh legumes, and lower consumption of pork, poultry or game, fish, and seafood. These three dietary patterns explained 23.25% of the variation in food intake.

**Table 3:** Factor loadings for three dietary patterns derived from CHNS 2004 through exploratory factor analysis

Food group	Dietary patterns		
	Traditional southern	Modern	Traditional northern
Rice	0.8348	—	—
Wheat flour	-0.5628	—	—
Wheat buns, breads	-0.4500	—	0.6636
Deep-fried wheat	-0.2276	—	—
Corn and coarse grain	-0.4666	—	—
Starchy roots and tubers	—	—	0.6599
Fresh legumes	0.3458	—	0.5020
Dried legumes	—	—	—
Legume products	—	—	—
Nuts and seeds	—	—	—
Fresh vegetables	0.2319	—	—
Pickled, salted, or canned vegetables	—	—	—
Fruits	—	0.6875	—
Pork	0.3070	—	-0.5684
Other livestock meat	—	—	—
Offals	—	—	—
Poultry and game	—	0.2710	-0.2069
Eggs and egg products	—	0.4335	—
Fish and seafood	0.2423	—	-0.2046
Animal-based milk and dairy products	—	0.7021	—
Alcoholic beverages	—	—	—

Factor loadings with weak correlations with identified factors whose absolute values were ≤0.20 are not shown

The prevalence rates of general and central obesity across quartiles of the three dietary patterns are shown in Table 4. Adults with higher scores for the traditional southern dietary pattern had lower prevalence rates of central and central obesity. By contrast, general and central obesity were more prevalent among adults with higher scores for the traditional northern dietary pattern.

**Table 4:** Prevalence rates of general and central obesity in 2015 across quartiles of dietary patterns in 2004 among Chinese adults from CHNS

	Dietary pattern quartiles			
	Quartile 1	Quartile 2	Quartile 3	Quartile 4
N	1051	1052	1052	1052
**Traditional southern**				
General obesity (%)	208 (19.8)	153 (14.5)	118 (11.2)	120 (11.4)
Central obesity (%)	537 (51.1)	463 (44.0)	395 (37.6)	376 (35.7)
**Modern**				
General obesity (%)	134 (13.2)	154 (14.4)	152 (14.2)	159 (15.1)
Central obesity (%)	411 (40.5)	431 (40.2)	444 (41.6)	485 (46.1)
**Traditional northern**				
General obesity (%)	117 (11.1)	145 (13.8)	162 (15.4)	175 (16.6)
Central obesity (%)	390 (37.1)	428 (40.7)	458 (43.5)	495 (47.1)

The relationship between dietary patterns and later general obesity, as assessed using a logistic model, is shown in Table 5. Dietary patterns were determined at baseline in 2004, whereas the participants’ obesity statuses were assessed in 2015. After adjusting for confounding factors, participants in the highest quartile (Q4) of the traditional southern dietary pattern had lower odds ratios of general obesity (OR = 0.50, 95%CI: 0.39, 0.65) than those in the lowest quartile (Q1). There was no significant relationship between the modern dietary pattern and general obesity (OR = 1.26, 95%CI: 0.96, 1.65, *P* = 0.123) in all models. Moreover, adults in Q4 of the traditional northern dietary pattern had higher odds ratios of general obesity than those in Q1 (OR = 1.61, 95%CI: 1.23, 2.10).

**Table 5:** Odds ratios and 95% confidence intervals for general obesity in 2015 across quartiles of dietary pattern scores

	Dietary pattern quartiles				
	Quartile 1	Quartile 2	Quartile 3	Quartile 4	*p* for trend
**Traditional southern**					
Model 1	1 (Ref)	0.69 (0.55, 0.87)^**^	0.52 (0.41, 0.67)^***^	0.51 (0.40, 0.65)^***^	< 0.001
Model 2	1 (Ref)	0.68 (0.54, 0.86)^**^	0.53 (0.42, 0.68)^***^	0.51 (0.40, 0.65)^***^	< 0.001
Model 3	1 (Ref)	0.68 (0.54, 0.87)^**^	0.53 (0.41, 0.68)^***^	0.50 (0.39, 0.65)^***^	< 0.001
Model 4	1 (Ref)	0.69 (0.54, 0.88)^**^	0.52 (0.40, 0.67)^***^	0.50 (0.39, 0.65)^***^	< 0.001
**Modern**					
Model 1	1 (Ref)	1.10 (0.86, 1.41)	1.09 (0.85, 1.40)	1.17 (0.91, 1.50)	0.251
Model 2	1 (Ref)	1.10 (0.86, 1.42)	1.10 (0.86, 1.42)	1.18 (0.92, 1.51)	0.218
Model 3	1 (Ref)	1.12 (0.87, 1.44)	1.11 (0.86, 1.44)	1.26 (0.97, 1.64)	0.111
Model 4	1 (Ref)	1.13 (0.87, 1.45)	1.11 (0.85, 1.44)	1.26 (0.96, 1.65)	0.123
**Traditional northern**					
Model 1	1 (Ref)	1.28 (0.98, 1.66)	1.45 (1.13, 1.87)^**^	1.59 (1.24, 2.05)^***^	< 0.001
Model 2	1 (Ref)	1.26 (0.97, 1.64)	1.43 (1.11, 1.85)^**^	1.58 (1.23, 2.03)^***^	0.001
Model 3	1 (Ref)	1.26 (0.97, 1.64)	1.42 (1.09, 1.84)^**^	1.57 (1.21, 2.04)^**^	< 0.001
Model 4	1 (Ref)	1.29 (0.98, 1.69)	1.40 (1.07, 1.83)^**^	1.61 (1.23, 2.10)^***^	< 0.001

Model 1: no adjustments, model 2: adjusted for age and gender, model 3: additionally adjusted for ethnicity, education level, marital status, work status, and residency based on model 2, model 4: additionally adjusted for physical activity, sleep duration, current smoking, alcohol consumption, and total energy intake per day based on model 3. Dietary patterns were identified at baseline in 2004. ^**^ *P*<0.01, ^***^ *P*<0.001

Unadjusted and adjusted odds ratios for later central obesity across the quartiles of dietary pattern scores are shown in Table 6. Similarly, we found that people in the highest quartile (Q4) of the traditional southern dietary pattern were less likely to develop central obesity (OR = 0.52, 95%CI: 0.43, 0.63) than those in the lowest quartile (Q1). For the modern dietary pattern, adults in Q4 were positively associated with central obesity (OR = 1.26, 95%CI: 1.06, 1.50), but this association was not significant after controlling for family, lifestyle, and energy intake covariates. Participants in Q4 of the traditional northern dietary pattern had a greater risk of developing central obesity than those in Q1 (OR = 1.64, 95%CI: 1.36, 1.98).

**Table 6:** Odds ratios and 95% confidence intervals for central obesity in 2015 across quartiles of dietary pattern scores

	Dietary pattern quartiles				
	Quartile 1	Quartile 2	Quartile 3	Quartile 4	*p* for trend
**Traditional southern**					
Model 1	1 (Ref)	0.75 (0.63, 0.89)^**^	0.58 (0.48, 0.68)^***^	0.53 (0.45, 0.63)^***^	< 0.001
Model 2	1 (Ref)	0.74 (0.62, 0.88)^**^	0.57 (0.48, 0.68)^***^	0.55 (0.46, 0.65)^***^	< 0.001
Model 3	1 (Ref)	0.72 (0.61, 0.87)^***^	0.58 (0.48, 0.69)^***^	0.56 (0.47, 0.67)^***^	< 0.001
Model 4	1 (Ref)	0.75 (0.63, 0.91)^**^	0.57 (0.47, 0.68)^***^	0.52 (0.43, 0.63)^***^	< 0.001
**Modern**					
Model 1	1 (Ref)	0.99 (0.83, 1.18)	1.05 (0.88, 1.25)	1.26 (1.06, 1.50)^*^	0.008
Model 2	1 (Ref)	1.00 (0.83, 1.19)	1.07 (0.90, 1.28)	1.29 (1.08, 1.54)^**^	0.003
Model 3	1 (Ref)	0.96 (0.80, 1.15)	1.03 (0.85, 1.23)	1.20 (0.99, 1.45)	0.040
Model 4	1 (Ref)	0.97 (0.81, 1.17)	1.02 (0.85, 1.22)	1.18 (0.97, 1.43)	0.084
**Traditional northern**					
Model 1	1 (Ref)	1.16 (0.98, 1.39)	1.31 (1.10, 1.56)^**^	1.51 (1.27, 1.79)^***^	< 0.001
Model 2	1 (Ref)	1.13 (0.95, 1.35)	1.25 (1.05, 1.49)^*^	1.49 (1.25, 1.78)^***^	< 0.001
Model 3	1 (Ref)	1.15 (0.96, 1.38)	1.26 (1.05, 1.51)^*^	1.57 (1.31, 1.88)^***^	< 0.001
Model 4	1 (Ref)	1.20 (0.99, 1.45)	1.33 (1.10, 1.60)^**^	1.64 (1.36, 1.98)^***^	< 0.001

Model 1: no adjustments, model 2: adjusted for age and gender, model 3: additionally adjusted for ethnicity, education level, marital status, work status, and residency based on model 2, model 4: additionally adjusted for physical activity, sleep duration, current smoking, alcohol consumption, and total energy intake per day based on model 3. Dietary patterns were identified at baseline in 2004. ^*^P<0.05, ^**^ *P*<0.01, ^***^ *P*<0.001

## Discussion

In the present study, we conducted an analysis of dietary patterns in Chinese adults aged 18–65 years at baseline and the association of dietary patterns with later general and central obesity. Three distinct dietary patterns, “traditional southern,“ “modern,“ and “traditional northern,“ were identified in this multicenter longitudinal study that satisfactorily captured eating habits, explaining 23.25% of the variance of dietary intake. These three dietary patterns were consistent with several studies previously conducted among samples of the Chinese population [37–40]. In addition, we found that the traditional southern dietary pattern was inversely associated with the risk of later general and central obesity after adjusting for all confounders. By contrast, the traditional northern dietary pattern was significantly related to an increased likelihood of developing subsequently general and central obesity.

The key components of the traditional Chinese diet include high consumption of grains, vegetables, legumes, and small amounts of animal-based foods [41]. Such a diet is low in fat and energy density and high in dietary fiber, which shares many similarities with the Mediterranean diet [42]. In the present study, traditional Chinese food has a high load on the factor of the southern and northern dietary patterns. In southern China, people prefer dishes combining rice, vegetables, and pork. However, northern Chinese are more likely to eat wheat flour-based staples such as steamed and stuffed buns. Therefore, we labeled these two dietary habits as traditional Chinese southern and northern dietary patterns.

We found that the traditional southern rice-based diet had a protective role on the subsequent development of general and central obesity. Generally speaking, rice is considered low-energy-density food because it absorbs more water when cooked than wheat flour [43]. Our findings are consistent with the results of a 5-year prospective study conducted in Jiangsu Province, China, showing that a traditional rice-enriched dietary pattern is associated with less weight gain [44]. However, the association between rice and weight status remains controversial in other Asian populations. In a nationally representative cross-sectional study of South Korea, white rice and kimchi dietary patterns were positively correlated with obesity [45]. A Japanese study also reported that a dietary pattern based on rice, miso soup, and soy products increased the risk of obesity [46]. The exact reason has not been clarified, but it may be due to differences in rice types and cooking methods in different regions or differences in nutrients such as starch types [39].

The protective role of the traditional southern diet on obesity may be attributed to dietary diversity and several other health-promoting foods. Fresh legumes, pork, and fish provide ample amounts of different types of protein for the rice staple pattern. Vegetables rich in dietary fibers are considered quite low-energy-density food, and studies have shown that high dietary fiber intake is associated with a reduced risk of obesity [47]. In addition, omega-3 polyunsaturated fatty acids (n-3 PUFAs) contained in fish regulate lipid metabolism and have protective role against weight gain [48].

In our study, the traditional northern dietary pattern was positively associated with the risk of general and central obesity later in life, which is consistent with several previous cross-sectional studies [37–39]. The northern dietary pattern, based on wheat, starchy tubers, and fresh legumes, has fewer food types and provides fewer micronutrients than the traditional southern dietary pattern. It is thought to represent a low-nutrient-density diet [40]. Evidence suggests that trace elements such as zinc, as well as vitamins A and C, play important regulatory roles in improving obesity and lipid metabolism in the body [49, 50]. Another characteristic of the traditional northern dietary pattern is the high proportion of carbohydrate intake. However, the mechanisms by which carbohydrates induce overweight and obesity remain hotly debated [51]. High carbohydrate intake might alter lipid profiles, such as increased triglycerides or decreased high-density lipoprotein cholesterol, leading to an increased risk of obesity [52]. Further research is needed to elucidate the underlying mechanisms.

The modern dietary pattern consisted of a combination of fruits, poultry or game, eggs or egg products, and animal-based milk and dairy products. We found no association between the modern dietary pattern with later development of weight status. Although the health benefits of fruits are well documented, the high concentration of simple sugars in certain fruits is thought to be one of the factors that contribute to weight gain [53]. The commercialization of food products has increased the availability of ready-to-eat fruits, such as canned fruits and fruit juices, and while extending shelf life, the significantly reduced dietary fiber content will reduce satiety and facilitate additional food intake [54]. In addition, an epidemiological study of populations from 10 European countries showed that poultry intake was associated with weight gain, which may be one of the reasons why no weight loss benefit was found in our study [55].

These findings may well have important implications for public health practice, not only for demonstrating the important roles of certain food groups on obesity in the Chinese adult population, but also for providing practical recommendations for future nutritional intervention programs. We proposed that increasing the frequency and intake of healthy food groups and increasing more varied eating patterns in nutrition practice to maintain appropriate energy intake may reduce the risk of obesity. Likewise, these findings could represent easy and effective public nutritional strategies for community dwellers who may not have regular access to nutritionally knowledgeable professionals or be able to prepare a variety of foods in full accordance with recommended healthy eating patterns, but advising them to eat a wider variety of healthy foods is simple advice that might be easy to put into practice.

We acknowledge several limitations in this study. First, this longitudinal study was clearly prone to high numbers of loss to follow-up and drop-outs, reducing the amount of data available at the later time points. This has resulted in a smaller sample size, reduced statistical power and thus increased likelihood of selection bias. Second, the 24-h dietary recall method was insufficient to assess the usual dietary intake and eliminate recall bias. Third, we had to make several subjective decisions to conduct this study; for example, classify foods into food groups, extract the number of factors, apply a rotation method, and label the factors. Fourth, we identified three major dietary patterns to capture the most typical eating habits of the Chinese population. However, some minor dietary patterns might also be associated with general and central obesity. In addition, due to changes in preferences and food availability, dietary patterns may change over time leading to some degree of misclassification that might affect the associations measured. Future studies on this topic should capture temporal trends in dietary patterns and obesity to better explain their association. Finally, although our analyses were controlled for multiple confounders, we cannot discount the possibility of residual confounding.

## Conclusions

In conclusion, in our sample of 4207 Chinese adults aged 18–65 years, we identified three distinct dietary patterns: the traditional southern pattern, modern pattern, and traditional northern pattern. The results of the present study indicate that the traditional southern dietary pattern is inversely associated with the risk of later development of general and central obesity, whereas the traditional northern dietary pattern is significantly related to an increased likelihood of developing general and central obesity. The current study provides longitudinal evidence for the association between dietary patterns and the development of obesity phenotypes among Chinese adults. Our findings might be useful to better guide the development of evidence-based preventive nutrition interventions to control the alarming obesity epidemic.

## Data Availability

The datasets generated and/or analysed during the current study are available from the official CHNS website (https://www.cpc.unc.edu/projects/china).
